# Prophylactic nimodipine treatment for hearing preservation after vestibular schwannoma surgery: study protocol of a randomized multi-center phase III trial—AkniPro 2

**DOI:** 10.1186/s13063-021-05417-z

**Published:** 2021-07-22

**Authors:** Christian Scheller, Christian Strauss, Sandra Leisz, Pia Hänel, Ariane Klemm, Simone Kowoll, Iris Böselt, Torsten Rahne, Andreas Wienke

**Affiliations:** 1 Department of Neurosurgery, Martin-Luther-University Halle-Wittenberg, Ernst-Grube-Str. 40, 06097 Halle (Saale), Germany; 2 Coordination Centre for Clinical Trials, Martin-Luther-University Halle-Wittenberg, Halle (Saale), Germany; 3 Department of Otorhinolaryngology, Head and Neck Surgery, Martin-Luther-University Halle-Wittenberg, Halle (Saale), Germany; 4 Institute of Medical Epidemiology, Biostatistics, and Informatics, Martin-Luther-University Halle-Wittenberg, Halle (Saale), Germany

**Keywords:** Nimodipine, Hearing preservation, Vestibular schwannoma, Acoustic neuroma

## Abstract

**Background:**

A previously performed phase III trial on 112 subjects investigating prophylactic nimodipine treatment in vestibular schwannoma (VS) surgery showed no clear beneficial effects on preservation of facial and cochlear nerve functions, though it should be considered that protection of facial nerve function was the primary outcome. However, the risk for postoperative hearing loss was halved in the nimodipine group compared to the control group (OR 0.49; 95% CI 0.18–1.30; p = 0.15). Accordingly, this phase III extension trial investigates the efficacy and safety of prophylactic nimodipine for hearing preservation in VS surgery.

**Methods:**

This is a randomized, multi-center, two-armed, open-label phase III trial with blinded expert review and two-stage with interim analysis. Three hundred thirty-six adults with the indication for microsurgical removal of VS (Koos I–IV) and serviceable preoperative hearing (Gardner-Robertson scale (GR) 1–3) are assigned to either the therapy (intravenous nimodipine 1–2 mg/h from the day before surgery until the fifth postoperative day and standard of care) or the control group (surgery only and standard of care). The primary endpoint of the trial is postoperative cochlear nerve function measured before discharge according to GR 1–3 versus GR 4–5 (binary). Hearing function will be determined by pre- and postoperative audiometry with speech discrimination, which will be evaluated by a blinded expert reviewer. Furthermore, patient-reported outcomes using standardized questionnaires will be analyzed.

**Discussion:**

Prophylactic parenteral nimodipine treatment may have a positive effect on hearing preservation in VS surgery and would improve patient’s quality of life. Further secondary analyses are planned. Except for dose-depending hypotension, nimodipine is known as a safe drug. In the future, prophylactic nimodipine treatment may be recommended as a routine medication in VS surgery. VS can be considered as an ideal model for clinical evaluation of neuroprotection, since hearing outcome can be classified by well-recognized criteria. The beneficial effect of nimodipine may be transferable to other surgical procedures with nerves at risk and may have impact on basic research.

**Trial registration:**

EudraCT 2019-002317-19, DRKS00019107. 8th May 2020.

## Background

Vestibular schwannomas (VS) are benign tumors, which comprise 6–8% of all intracranial tumors with an overall incidence of 1–2/100,000/year [1]. The most prevalent symptoms in patients suffering from VS are hearing loss, tinnitus, and vertigo. Disease management options include follow-up by magnet resonance imaging (MRI), stereotactic radiosurgery, and microsurgical removal.

However, the goal of modern VS surgery is total tumor removal with preservation of facial and cochlear nerve function. Deterioration of cochlear nerve functions is a common complication following VS surgery. Except for dexamethasone, there is a lack of neuroprotective medication in neurosurgical interventions. Nimodipine, a dihydropyridine calcium antagonist, is a generally well-tolerated drug with a long history in neurosurgical practice. Besides preventing cerebral vasospasm, a neuroprotective effect has been assumed [2]. Further, basic research points to an underlying neuroprotective mechanism of nimodipine [3–6]. Herzfeld et al. demonstrated a reduction of apoptosis in nimodipine-treated neuro2a cells via surgery-like stress models [4, 5]. Several monocenter, retro- and prospective clinical trials pointed to a beneficial effect of nimodipine on the long-term outcome of cranial nerve functions following VS surgery [7–14]. A monocentric pilot study on 30 subjects showed a superiority of its prophylactic administration compared to an intraoperative start or no treatment [9]. A subsequently performed phase III trial on 112 subjects with facial nerve function 12 months after surgery as primary outcome showed no clear beneficial effects [15]. But, the risk for postoperative hearing loss was halved in the treatment group compared to the control group (OR = 0.49; 95% CI 0.18–1.30; p = 0.15), particularly in medium- to large-sized tumors (Koos III/IV) [15]. However, a subsequently performed combined analysis of the phase III trial and its pilot study showed significantly lower risks for hearing loss in the treatment group, for all tumor sizes (OR = 0.38; 95% CI 0.17–0.83; p = 0.016) [16]. In Germany, nimodipine is occasionally used in VS surgery (off-label use); however, sufficient evidence by randomized controlled trials of a clear neuroprotective efficacy is still missing. Therefore, the main aim of the study is to clarify whether prophylactic nimodipine preserves hearing in vestibular schwannoma surgery.

## Methods/design

### Objectives

This trial investigates the efficacy and safety of prophylactic nimodipine for hearing preservation in VS surgery.

Preservation of hearing using prophylactic nimodipine would significantly improve the patient’s quality of life. In the future, prophylactic nimodipine treatment may be recommended as a routine medication in VS surgery. VS can be considered as an ideal model for clinical evaluation of neuroprotection, since the hearing outcome can be classified by well-recognized criteria (Gardner-Robertson scale (GR)) [17]. The primary objective is to assess the effect of prophylactic parenteral preoperatively starting nimodipine treatment for hearing preservation after VS surgery in comparison to standard care only. This will be measured by pure-tone and speech audiometry during the postoperative hospital stay. The primary endpoint is postoperative cochlear nerve function measured before discharge according to GR scale 1–3 versus GR 4–5 (binary), a suitable predictor for the preservation of serviceable hearing. The beneficial effect of nimodipine may be transferable to other surgical procedures with nerves at risk.

### Trial design

The trial will be randomized two-armed, open-label, with blinded expert review and interim analysis.

## Methods: participants, interventions, and outcomes

### Study setting

The clinical trial will be carried out in a multi-center fashion at currently 8 trial sites in Germany—mainly academic hospitals as well as a community clinic; for a list, refer to https://www.drks.de (ID DRKS00019107). The participating sites have a large expertise in VS surgery and also in nimodipine treatment. All sites are experienced in conducting GCP-conform clinical trials. Several trial sites had already participated in the previous multi-center trial [15]. Additional qualified trial sites can be included during trial conduct if necessary.

For data management, monitoring, safety management, biostatistics, and project management, the Coordinating Investigator will be supported by the Coordination Centre for Clinical Trials (CCT/KKS Halle), which has comprehensive experience with drug trials. This trial with its protocol is approved by the German Competent Authority and the Ethics Committees of each site and is registered at https://www.drks.de (ID DRKS00019107).

### Eligibility criteria

Patients with VS, indication for surgery, and preoperative serviceable hearing (GR 1–3) will be enrolled. Based on the data of both previous trials [9, 15, 16], patients suffering from VS of all tumor sizes will participate. However, patients with neurofibromatosis and previous therapy (surgery and/or radiotherapy) are excluded.

#### Inclusion criteria

1. Adults age ≥ 18 years
2. VS (Koos I–IV) with indication for surgery
3. Preoperative pure-tone audiogram (not older than 3 months prior to surgery), hearing function according to GR 1–3 (air conduction pure-tone average (PTA) (0.5–3 kHz) < 90 dB hearing level (HL))
4. Preoperative MRI (not older than 6 months prior to surgery)
5. Written informed consent obtained according to international guidelines and local laws
6. Ability to understand and give informed consent
7. Safe contraception measures for males and females

#### Exclusion criteria

1. Hearing function GR 4–5 (air conduction PTA (0.5–3 kHz) ≥ 90 dB HL)
2. Conductive hearing loss at the affected side (mean air bone gap (0.5, 1, 2, 3 kHz) > 10 dB) if bone conduction is measurable
3. Previously irradiated or surgical treated VS
4. Neurofibromatosis, other brain tumors, other reasons for inoperability
5. Pregnancy and lactation period
6. Known hypersensitivity to nimodipine or any of the excipients (ethanol, Macrogol, sodium citrate, citric acid) to be used for nimodipine infusion
7. History of (reformed alcoholics) or persistent abuse of alcohol
8. Known current kidney or liver insufficiency
9. Any medical condition that in the opinion of the investigator would not permit participation in the clinical trial
10. Unstable angina pectoris and/or myocardial infarction during the last 4 weeks before the start of treatment
11. Severe, uncontrolled, symptomatic hypotension at inclusion
12. Subjects with psychological, psychiatric, neurological, familial, sociological, or geographical conditions that do not permit compliance with the protocol as well as officially and/or legally accommodated persons
13. Participation in another interventional trial simultaneously and within the last 30 days prior to inclusion (registries or observational studies allowed)

### Discontinuation criteria and dropouts

Patients can have treatment or trial participation terminated prematurely at any time, without having to give reasons. If the patient agrees, the visits, follow-up, and documentation will continue as scheduled. Further reasons for discontinuation are the occurrence of adverse events related to the study treatment that cannot be treated adequately or of adverse events which in the opinion of the investigator make it necessary to stop the study treatment. The investigator may also terminate the study participating for the patient in case of missing compliance or other circumstances which make trial-relevant follow-up impossible. The sponsor might also decide to withdraw a patient from trial participation (the reason has to be specified, e.g., major protocol violation).

If a patient decides to discontinue the study after randomization but before the respective start of treatment, the patient will be considered as a dropout.

### Interventions

Patients will be randomized and assigned to one of two groups (interventional or control group).

#### Control group

Patients in the control group will receive surgery only, as the standard of care, and be prepared, operated, and treated afterwards as is required by routine procedures at the trial site. Nimodipine administration is not allowed in control patients for the whole trial duration except in case of emergency of aneurysmal subarachnoid hemorrhage (SAH).

#### Interventional group

Patients assigned to the interventional group will receive nimodipine parenterally from the day before surgery until the 5th postoperative day additional to surgery as standard care. Nimodipine should be given as soon as possible on the day before surgery. However, due to the clinical situation, a defined time was not determined. Administration of nimodipine will be started via a peripheral venous access with a dosage of 1 mg/h (5 ml/h) for at least 2 h. If nimodipine is well tolerated, the dosage should be increased to 2 mg/h (10 ml/h). During and after surgery, nimodipine should be administered via a routine central line. Blood pressure should be monitored three times a day due to the effects of and on nimodipine. In case of hypotension, a dose reduction of 1 mg/h or 0.5 mg/h or a stop of treatment is intended. The administration of nimodipine and thus the cumulative dosage will be documented for each patient. Afterward, until the patient’s end of study, nimodipine treatment is not permitted except in case of emergency of aneurysmal SAH.

Nimodipine will be purchased by Bayer Vital GmbH, Leverkusen, Germany.

#### Treatment modification

Dose reduction is essential in the case of symptomatic patients caused by hypotension, e.g., dizziness and fatigue. An initial dose reduction from 2 mg/h (10 ml/h) to 1 mg/h (5 ml/h) is recommended. Thereafter, the dose should be reduced to 0.5 mg/h (2.5 ml/h) if the patient is still symptomatic. Nimodipine treatment may also be discontinued. However, dose reduction/discontinuation due to other adverse events as well as rechallenge of nimodipine will be the investigator’s discretion. All dosages administered to the patient and any dose change or interruption will be recorded.

### Outcomes

#### Primary outcome

The primary endpoint is postoperative cochlear nerve function measured before hospital discharge according to GR 1–3 versus GR 4–5 (binary). This outcome defines the preservation of useful hearing.

#### Secondary outcomes

The secondary endpoints are the following:

- Late postoperative cochlear nerve function measured at follow-up 3 to 6 months after surgery according to GR 1–3 versus GR 4–5 (binary)
- Postoperative deterioration of cochlear nerve function of at least one grade according to the GR compared with its preoperative function (binary)
- Late postoperative deterioration of cochlear nerve function of at least one grade according to the GR compared with its preoperative function measured at follow-up 3 to 6 months after surgery (binary)
- Change of pure-tone thresholds (PTA) and speech discrimination score (Word Recognition Score, WRSmax)
- GR and American Academy of Otolaryngology-Head and Neck Surgery (AAOHNS) scores
- Intraoperative deterioration of brainstem audiometry evoked potential (BAEP) mean values (amplitudes and latencies)
- Subjective quality of life/psychosocial impairment will be assessed by the means of three patient’s questionnaires (Hearing Handicap Inventory for the Elderly (HHIE), Short Form (SF)-12, Penn Acoustic Neuroma Quality of Life (PANQOL)) before surgery and at follow-up
- Anatomical preservation of the cochlear nerve and preservation of wave V (BAEP)
- Safety data

### Participant timeline

Duration of intervention: 7 days

Duration of follow-up: 3 to 6 months

Study related: estimated start 4th quarter 2020. Duration of enrolment: 21 months, interim analysis, 21 months. Estimated end of the trial: 2025

**Fig. 1:**
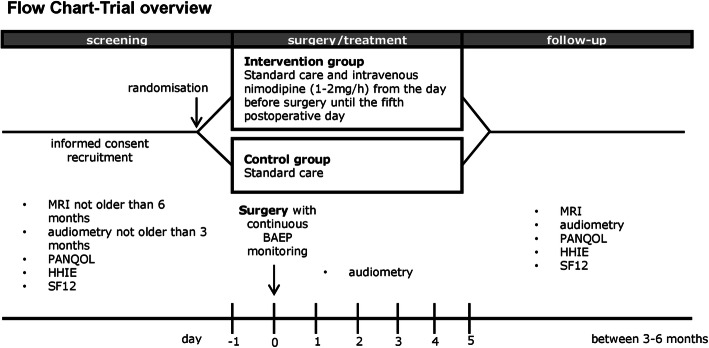
Flow chart

**Fig. 2:**
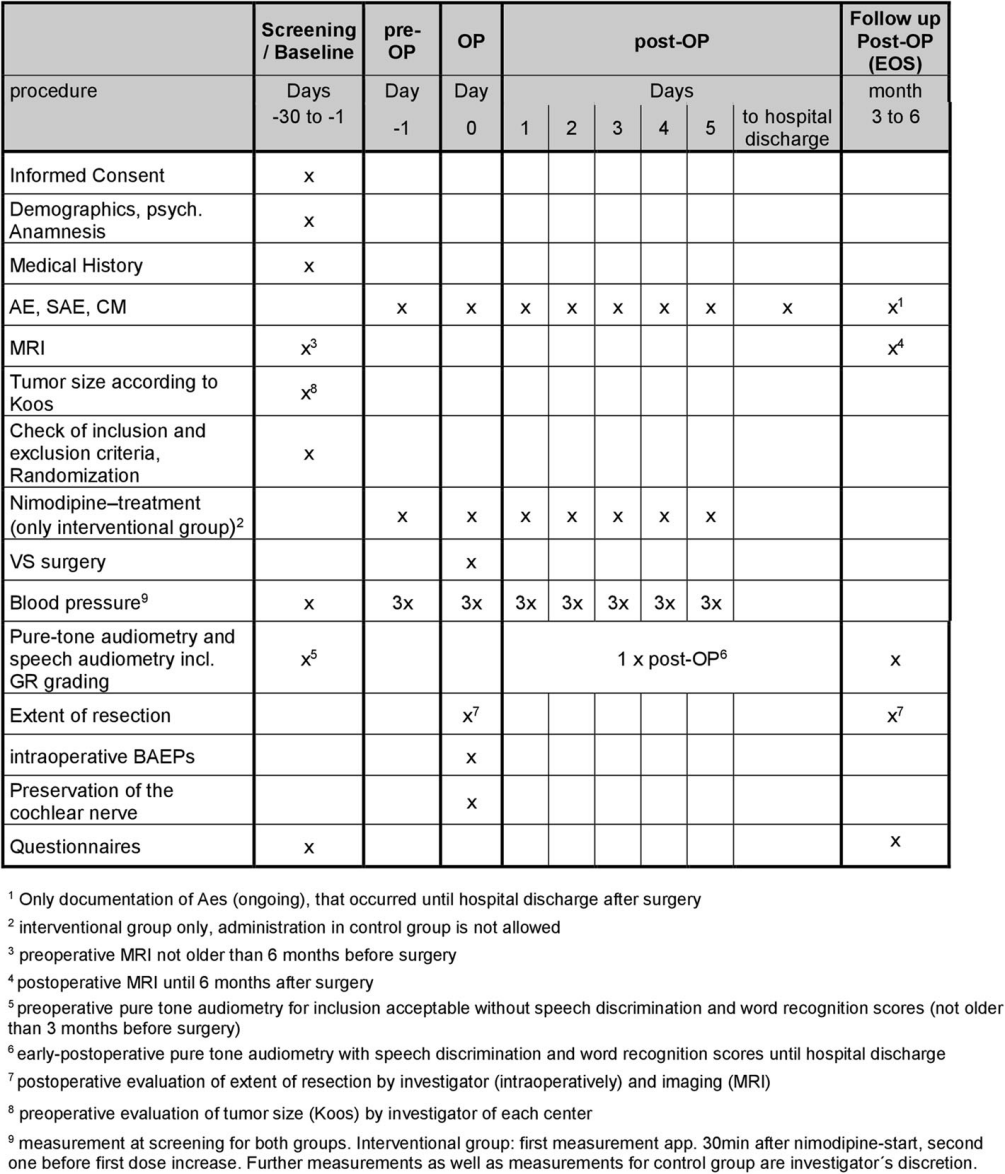
Visit schedule and assessments

A detailed visit schedule and trial flow chart is provided (Figs. 1 and 2). The schedule lists all of the evaluations in tabular form and indicates with an “X” when they have to be performed.

### Sample size

The sample size calculation is based on the combined results of the pilot study and the multi-center study regarding the primary outcome postoperative cochlear nerve function [9, 15, 16]. Considering patients with tumor size Koos I–IV and preoperative hearing function GR 1–3, 30 out of 46 patients (65.2%) suffered from deaf in the control group versus 31 out of 63 patients (49.2%) in the therapy group. Using these frequencies, based on a two-sided chi-square test with alpha = 5% and 80% power and an interim analysis at information fraction 0.5 with O’Brien-Fleming-type alpha-spending and possible trial stop because of futility, requires 80 patients per group in the first stage. Additional 79 patients per group are necessary if the trial is not stopped because of futility after the interim analysis (SAS® procedure SEQDESIGN). Assuming a dropout rate of 5%, the planned sample size for the trial is 84 patients per treatment group in the first stage and 84 additional patients per group in the second stage. Finally, the total maximum number of patients required for both stages is 336 (168 before interim analysis and 168 after interim analysis).

### Recruitment

Each site will screen their VS patients with indication for surgery for recruiting. Inclusion of all tumor sizes (Koos I–IV) [18] and the close patient-physician relationship in this indication will facilitate recruitment. To date, 4 study centers which have already participated in the previous study with high recruitment rates and 4 additional trial sites with good VS surgery and clinical trial reputation will participate. All sites had committed their planned recruitment via feasibility questionnaires. In the previous trial [15], recruitment was completed even faster than planned, so recruitment is expected to be feasible again without delay.

## Methods: assignment of interventions

### Allocation

To ensure concealment of treatment allocation, stratified block randomization with respect to tumor size (Koos I/II vs. III/IV) and variable block length will be performed centrally in SAS and included in SecuTrail for Internet-based online randomization independently organized by CCT Halle.

### Blinding

A double-blind design, usually the favorable approach in clinical research, has not been proven as feasible for several reasons. From an ethical point of view, a double-blind approach using a placebo would require a central-line catheter for 5 days in the postoperative course in patients of the control group. Furthermore, manufacturing of an appropriate i.v. placebo is not feasible.

## Methods: data collection, management, and analysis

### Data collection methods

#### Tumor size and extent of resection

Tumor size is determined by MRI (thin-slice, axial T1-weighted with and without contrast enhancement, not older than 6 months prior to surgery) and classified according to Koos grading system [18] by the investigator of each trial site (Koos I/II vs. III/IV) for the stratified randomization. For statistical analysis, it is additionally evaluated by a blinded independent expert. The extent of resection will be evaluated by the investigator’s subjective impression intraoperatively and by an independent expert via MRI.

#### Audiometry/GR evaluation

Standard pure-tone audiometry provides diagnostic information concerning the degree, type, and configuration of hearing loss. Audiometries should be performed in a soundproof booth. Parameters are defined in International Organization for Standardization (ISO) norm (ISO 11957:1996, 1996). Speech audiometry is performed to obtain the speech recognition threshold by using German multisyllabic words (“Freiburger Zahlentest”), if possible, and suprathreshold speech recognition by using German monosyllabic words (“Freiburger Einsilbertest”), if possible. The following information should be provided additionally regarding the performed pure-tone and speech audiometries for both ears in each patient:

- Pure-tone thresholds for air conduction at frequencies of 0.25, 0.5, 1, 2, 3, 4, and 8 kHz. Masking has to be applied if needed.
- Pure-tone thresholds for bone conduction at frequencies of 0.5, 1, 2, 3, 4, and 6 kHz. Masking has to be applied if needed.
- The graph plotted by connecting all hearing threshold values for all tested frequencies as well as masking levels used.
- Speech reception threshold (SRT) for air conduction (ipsilateral headphone-presentation, “Freiburger Zahlentest”).
- If SRT < 65 dB sound pressure level (SPL): word recognition score for Freiburger Monosyllables at 65, 80, and 95 dB SPL, using 2 lists each, masking levels have to be reported.
- If SRT ≥ 65 dB SPL and SRT < 80 dB SPL: word recognition score for Freiburger Monosyllables at 80 and 95 dB SPL, using 2 lists each, masking levels have to be reported.
- If SRT ≥ 80 dB SPL: word recognition score for Freiburger Monosyllables at 95 dB SPL, using 2 lists, masking levels have to be reported.

GR evaluation: Hearing class is determined according to the GR grading system [17] by the investigator of each trial site for inclusion. If speech discrimination (WRS_max_) is not available, evaluation will be done by pure-tone measurements only. For statistical analysis, it is additionally evaluated by an independent expert. AAOHNS evaluation will only be done by a blinded expert [19].

If during surgery the nervus cochlearis has been cut, grading is considered as GR 5. In this case, postoperative audiometries do not have to be performed.

#### Questionnaires

Patients are asked for an evaluation of subjective health and communication competencies (quality of life) via three different patient questionnaires (SF-12 [20, 21], PANQOL [22], HHIE [23, 24]) twice during the trial: before surgery and during follow-up after 3 to 6 months. Interpretation of questionnaires and calculation of scores is highly standardized but will nevertheless be done by a blinded expert.

### Data management

All data will be entered directly at the trial site by the study team via remote data entry in the trial-specific electronic Case Report Form (eCRF). The study management software secuTrial®, a Good Clinical Practice (GCP)-compliant clinical data management system for the conduction of clinical trials, will be used for data entry and query management. Management of the study data base will be done by CCT Halle.

In a multistage procedure, the obtained data will be checked electronically for their plausibility and consistency by CCT Halle. In case of implausible or missing data, queries will be generated and provided to the investigator in order to obtain any missing information and resolve inconsistencies.

After completion of data capture and quality check, data base will be closed by CCT Halle and the data will be transferred to the biometrician for statistical analysis.

### Statistical methods

#### Populations for analysis

The primary analysis data set is the modified intention-to-treat population. This data set contains all the patients who have been enrolled in the clinical trial, randomized, and treated by surgery with pre- and postoperative values of the nervus cochlearis and tumor size.

The secondary analysis data set is derived from the per-protocol population. This data set includes all patients of the primary analysis data set without major inclusion/exclusion violations who have been treated according to their randomization schedule: For patients in the nimodipine group, this requires nimodipine treatment at least 18 h per day (with any dosis) from day −1 (day before surgery) until day 5 after surgery (in total 7 days); patients in the control group having received nimodipine will be excluded.

The tertiary analysis data set (safety population) contains all the patients who underwent surgery and/or received at least one dose of the study medication.

#### Evaluability of a subject

Patients are evaluable if pre- and postoperative values of the nervus cochlearis and the tumor size are available.

#### Final analysis

##### Primary endpoint

The primary efficacy analysis will be performed according to the intent-to-treat principle. Because of the expected very small number of dropouts (less than 5%), missing outcomes will be excluded from the analysis. To test the superiority of the intervention versus the control group, logistic regression will be used with the treatment group and tumor size (because of stratified randomization w.r.t. tumor size) as variables. Results will be presented as odds ratios including 95% Wald confidence intervals and p-values.

##### Secondary endpoints

Additional sensitivity analysis will be performed by a per-protocol analysis for the primary endpoint. The risk difference of postoperative hearing deterioration will be supplemented by a 95% Wald confidence interval, and the Mantel-Haenszel estimation (with tumor size according to Koos as strata variable) will be performed as a sensitivity analysis as well as an additional per-protocol analysis. The secondary outcome postoperative deterioration of cochlear nerve function of at least one grade according to the GR compared with its preoperative function will be analyzed similar to the primary outcome and results will be given as odds ratios including 95% Wald confidence intervals and p-values. Deterioration of BAEPs amplitudes and latencies of the intraoperative BAEP will be evaluated by comparing their differences between beginning and end of surgery using linear regression with covariates treatment group and tumor size (Koos I/II or Koos III/IV). Change in quality of life (SF-12, PANQOL, HHIE) will be also evaluated using linear regression with covariates treatment group and tumor size (Koos I/II or Koos III/IV). All analyses of secondary endpoints will be interpreted in an exploratory sense.

##### Safety

Safety data will be evaluated descriptively, including all randomized study patients, who received at least one dose of study medication and/or underwent surgery (safety population).

##### Methods for additional analyses, such as subgroup analyses and adjusted analyses

Additionally, logistic regression analyses of the primary endpoint adjusted for tumor size (Koos classification and volumetry), preoperative hearing, extent of resection, positioning of the patient during surgery (semi-sitting or supine), total dose of nimodipine, dose modification (yes/no), and intraoperative events, e.g., bleedings (yes/no), as well as subgroup analyses by the following subgroups are planned:

- Preservation of cochlear nerve intraoperative by surgeons’ statement (yes/no)
- Cystic tumor by blinded expert evaluation (yes/no)
- Sex
- BAEP (amplitude and latency: stable/worsened/totally lost)

## Methods: monitoring

### Data monitoring

As a measure of quality control, risk-adapted on-site data monitoring is conducted during the enrolment period by independent clinical monitors from the CCT Halle to ensure patient safety, adherence to protocol and GCP, and consistency of the data. This is done according to GCP and standard operating procedures (SOP). The specific extent of the monitoring and source data verification is specified in the monitoring manual. In addition, regular central data monitoring via electronic CRF will be performed during the study course to check the data quality in a continuous manner. Every trial site will undergo a close-out visit by the monitors after the last participant at that site has finished the follow-up. To ensure the quality of data and study performance, the sponsor may conduct site visits by an independent auditor.

An independent Data Safety Monitoring Board (DSMB) is established to monitor safety data during the course of the clinical trial. The composition (neurosurgeon, ENT, biostatistician) and responsibilities of the DSMB and the structure and procedures of its meetings are laid down in the DSMB charter.

#### Interim analysis

An interim analysis is planned for the time point when half of the patients (168 patients) have been randomized and their primary endpoint has been evaluated. The study design includes a two-stage design with interim analysis at information fraction 0.5 with O’Brien-Fleming-type alpha-spending and possible trial stop because of futility.

### Harms

Adverse events, e.g., any untoward medical occurrence in a subject without regard to the possibility of a causal relationship, occurring from the day before surgery until hospital discharge will be captured according to GCP and common standards. Any event meeting criteria of a serious adverse event will additionally be immediately reported to the sponsor according to legal regulation. Further expedited reporting to the competent authority, the relevant ethics committee, and all investigators are done in case of a suspected unexpected serious adverse reaction. For classification of the adverse events, the MedDRA code system will be used.

Any pregnancy that occurs during trial participation must also be reported.

### Auditing

In compliance with the International Conference on Harmonization (ICH)-GCP guidelines, audits may be performed as a quality measure by the sponsor or an independent external party, as well as inspections by regulatory authorities. For quality control by regular monitoring, please refer to the “Data monitoring” section.

### Ancillary and post-trial care

For all patients participating in this trial, an insurance covering trial-related harms is contracted according to national law. Post-trial care of VS will be the investigator’s discretion and done according to the German treatment guidelines/clinical routine.

#### Protocol amendments

Substantial amendments to the protocol according to definitions in national law have to be submitted by the sponsor and to be approved by the Competent Authority and Ethics Committees before being implemented. Only changes of the protocol that are required for patient safety may be implemented prior to approval.

### Dissemination policy

The trial is registered with full description at the German Clinical Trials Register (Deutsches Register Klinischer Studien, DRKS).

The results of the trial will be published independent of the size or direction of the effects, according to CONSORT guidelines, in peer-reviewed national and international journals with a special emphasis on media that is relevant for professionals in Neurosurgery. Additionally, clinical trial summary results will be published in the European Union Clinical Trials Database (EudraCT).

## Discussion

This randomized, two-armed, multi-centric phase III trial will investigate the efficacy and safety of prophylactic nimodipine for hearing preservation in VS surgery. The treatment in the control group is active standard medical care. Nimodipine is primarily used in SAH for more than 20 years. Except for dose-depending hypotension, nimodipine is a safe drug. Severe harms to the participants are not expected; however, all patients undergo a comprehensive clinical monitoring during trial participation including 3 to 6 months of follow-up after the end of study treatment. Thus, the potential benefit of the prophylactic treatment for hearing preservation and thus communication abilities in the professional and private life of the participants is considered to be much higher than the risk of potential side effects.

In principle, prophylactic treatment with neuroprotective drugs prior to interventions with nerve tissue at risk seems to be a novel and promising concept and may have influence on basic research (investigation of the underlying molecular mechanisms). The patients of the control group will not receive neuroprotective nimodipine treatment. In the first phase III trial, an intraoperative start of the medication was allowed in the control group [15]. This might be the reason for the missing significant effect of the treatment. In the clinical course, nimodipine is occasionally given as an off-label-use medication; however, the medication is not being applied at many neurosurgical departments in Germany. There are no recommendations for perioperative nimodipine treatment in the guidelines for VS surgery; thus, the treatment of the control group represents the international standard of care. Therefore, the approach with different treatments (preoperative nimodipine versus no medication) in the intervention and the control group is justified from an ethical point of view as well as to address the scientific question in an appropriate way. A double-blind design, usually the favorable approach in clinical research, has not been proven as feasible for the reasons described in the “Blinding” section.

Therefore, nimodipine will be administered parenterally via a routine central line from the day before surgery until the fifth postoperative day exclusively in the treatment group. The chosen dosage of 1–2 mg/h is the standard dosage of nimodipine in the treatment of aneurysmal SAH patients.

Since it has been shown that there is no significant change in cochlear nerve function between the early postoperative course and 1 year after VS surgery [25], a potential hearing loss from fluid in the mastoid/middle ear in the early postoperative course should not affect the substance of the findings.

Prophylactic parenteral nimodipine treatment may have a positive effect on hearing preservation in VS surgery and would significantly improve patient’s quality of life. Additionally, social and rehabilitation costs may be reduced when preservation of hearing can be achieved by prophylactic neuroprotective treatment. Therapy costs of nimodipine treatment are assumed to be considerably lower compared to individual rehabilitation measures on patients suffering from reduced hearing or even hearing loss. In the future, prophylactic nimodipine treatment may be recommended as a routine medication in VS surgery and possibly in other surgical procedures with nerves at risk.

## Trial status

Protocol version: AkniPro 2 V03F July 13, 2020

Study dates: Recruitment phase: January 2021 to January 2025 (planned)

Follow-up phase: May 2021 to May 2025

## Data Availability

All study-related information will be stored securely at the study site. Each trial site will have access to their eCRF data and receive a copy after closed data base. The sponsor has access to the complete data set of the trial. The regulations of the Data Protection laws will be complied with. It will be assured by the sponsor that all investigational materials and data will be pseudonymized before scientific processing.

## Abbreviations

AAOHNS: American Academy of Otolaryngology-Head and Neck Surgery
BAEP: Brainstem audiometry evoked potential
CCT Halle: Coordination Centre for Clinical Trials, University of Halle (Saale)
CONSORT: Consolidated Standards of Reporting Trials
DRKS: Deutsches Register Klinischer Studien
DSMB: Data Safety Monitoring Board
eCRF: Electronic Case Report Form
ENT: Ear, nose, and throat
EudraCT: European Union Clinical Trials Database
GCP: Good Clinical practice
GR: Gardner-Robertson scale
HHIE: Hearing Handicap Inventory for the Elderly
ICH: International Conference on Harmonization
ID: Identification
ISO: Organization for Standardization
i.v.: Intravenous
MRI: Magnet resonance imaging
PANQOL: Penn Acoustic Neuroma Quality of Life
PTA: Pure-tone average
SAH: Subarachnoid hemorrhage
SPL: Sound pressure level
SRT: Speech reception threshold
SF: Short Form
VS: Vestibular schwannoma
WRSmax: Word Recognition Score
